# Effect of Acute and Chronic Aerobic Exercise on Immunological Markers: A Systematic Review

**DOI:** 10.3389/fphys.2019.01602

**Published:** 2020-01-24

**Authors:** Ciro Alexandre Mercês Gonçalves, Paulo Moreira Silva Dantas, Isis Kelly dos Santos, Matheus Dantas, Daliana Caldas Pessoa da Silva, Breno Guilherme de Araújo Tinoco Cabral, Ricardo Oliveira Guerra, Geraldo Barroso Cavalcanti Júnior

**Affiliations:** ^1^Graduate Program in Health Sciences, Federal University of Rio Grande Do Norte, Natal, Brazil; ^2^Graduate Program in Physical Education, Federal University of Rio Grande Do Norte, Natal, Brazil

**Keywords:** immune markers, immune system, physical activity, leukocytes, lymphocytes

## Abstract

**Introduction:** The effects of aerobic exercise on the immune system are not yet fully defined in the scientific literature. This fact demonstrates the need to investigate its influence on existing immunological markers by classifying and quantifying their acute and chronic effects.

**Objective:** To investigate the effects of acute and chronic aerobic exercise on inflammatory markers of healthy adults.

**Methods:** This study is a systematic review according to PRISMA recommendations. The following databases were searched: MEDLINE (via PubMed), Science Direct, Scopus, Web of Science, SciELO, Bireme and Cochrane Library, and article references. The last search was performed in March 2019. We included randomized controlled trials (RCTs) and non-randomized controlled trials (NRCTs) investigating the acute and chronic effects of aerobic exercise on immune markers in healthy male and female adults aged 20 to 45 years, without restrictions in language or year of publication. Two authors independently analyzed the studies by reading the titles, abstracts, and full texts. Risk of Study bias was analyzed using Cochrane's Risk of Bias Tool.

**Outcomes:** We included 15 studies in this systematic review, 13 of which were acute intervention and 2 were chronic, with 296 participants, 196 men and 100 women all being healthy individuals. It was observed that the acute intervention promotes changes in most immunological markers, while the chronic intervention interferes with a smaller proportion, this being in lymphocyte subpopulations. In the evaluation of quality, it was found that most studies did not present a high risk of bias in the evaluated aspects, but an unclear related risk of bias was observed, requiring a more careful analysis.

**Conclusion:** Thus, it can be concluded that the evidence indicates that acute and chronic interventions may modify most immune markers, but aspects such as gender, contraceptive pill use in women, physical capacity of the investigated individuals, environment, and type and intensity of the exercises may interfere with these markers as well as the data analysis. Therefore, this review suggests that further research is needed to contribute to the confirmation and estimation of results.

## Introduction

The immune system (IS) is a complex interaction between cells and molecules that acts to protect the host against possible microorganism invasions, prevent disease and enable wound healing (Simpson et al., 2015). The system itself is divided into 2 major groups that act synergistically on the overall immune response: The innate system (phagocytes and natural killer cells) and the adaptive system (B and T cells). Communication between defense systems is performed through cytokines and other messengers (Berger, 2000; Raphael et al., 2015), which interact to produce defense responses.

Interestingly, physical exercise has been shown to be a stressor capable of promoting an acute breakdown of the IS stable state and promoting chronic adaptations (Córdova et al., 2010). Aerobic exercise itself is a potential disruptor of the cells of healthy athletes (Nieman et al., 1995), as it can be structured into different volumes and intensities (Hofmann and Tschakert, 2017). Continuous aerobic exercise practice is likely to induce an increase in leukocytes, cytokines, interleukins, and tumor necrosis factor alpha in blood serum (Andersson et al., 2010). Adaptive immunity is not significantly altered by athletic exertion, but the innate system responds differently to chronic stress by increasing natural killer cell activity and decreasing neutrophil action (Nieman and Pedersen, 1999).

Several immunological markers change after long periods of physical exertion allowing the “open window” phenomenon to occur for 3 to 72 h which decreases immunity and provides a greater onset of airway infections (Pedersen and Ullum, 1994; Nieman and Pedersen, 1999; Gleeson, 2007). This impact of exercise is well-characterized when it is analyzed in conjunction with the occurrence of upper airway infections. Exercising at different intensities and the subject's level of physical fitness directly interfere with the immune system response, as shown in the explanatory model of the “J” shaped curve (Nieman, 1994). Exercise can induce hormonal changes, such as increased cortisol levels (Wang et al., 2019). These changes are described as mediating immunosuppression after the training session (Smith, 2004).

Despite the existence of evidence on the effects of physical exercise on the immune system of trained individuals, cyclists, triathletes and marathon runners, the acute and chronic responses brought on by aerobic exercise need to be investigated due to the existence of omissions related to the recommendations that stimulate changes in the immunological markers (Nieman et al., 2001; Brown et al., 2015; Barros et al., 2017; Koh and Park, 2018). This information is important to clarify the role of aerobic exercise in immune markers and to suggest new evidence that will contribute to the prescription of aerobic training in inducing different immune responses. This systematic review aims to investigate the effects of acute and chronic aerobic exercise on immune markers in healthy individuals.

## Methods

### Search Strategy

This systematic review was performed following the guidelines and recommendations of the PRISMA Systematic Review and Meta-Analysis Preferred Report items (Moher et al., 2009). The protocol of the review has been registered in the Prospective Registry (PROSPERO) data file under the registration number: CRD42017058899.

The studies were searched for in the following databases: MEDLINE (via PubMed), Science Direct, Scopus, Web of Science, SciELO, Bireme and Cochrane Library. The following search strategies, terms (MESH), and Boolean operators were considered: “physical activity” OR exercise OR training AND “Immune System” OR “immune function” OR “immune cell” AND “killer cell” OR “t cell” OR “cytokine^*^” OR “interleukin^*^” OR “leukocyte^*^” OR “lymphocyte^*^” OR “adhesion molecule” AND “adult^*^” OR “human^*^”. The last search was performed in March 2019. Two authors (CAMG, IKS) independently reviewed the titles and abstracts (Level 1). Subsequently, full versions of the articles that met the inclusion criteria were obtained (Level 2). After the analyzes, the reference list of the articles that met the criteria was analyzed to identify additional studies. The study analyzes were resolved with the help of a third author (MPD), duplicate studies with lack of content and access (after sending an email to the authors requesting more information) were excluded.

### Inclusion and Exclusion Criteria

Inclusion criteria were as follows: randomized controlled trials (RCTs) and non-randomized controlled trials (NRCTs), interventions that use acute or chronic aerobic exercise, which analyze certain markers of the immune system, and healthy adults of both sexes aged between 20 and 45 years. This age group excluded adolescent individuals and menopausal women due to immunological interference (Giefing-Kröll et al., 2015; Chen et al., 2016; Brazil Ministry of Health, 2018). There were no restrictions for language or year of publication in the inclusion of the articles. Studies with adult smokers, pregnant women, patients undergoing any type of cardiac treatment, and patients with any type of immune system disorder (e.g., immunodeficiencies, inflammatory and autoimmune diseases) were excluded. Other studies that were excluded were: unpublished data, observational studies, review articles, studies using other types of neuromuscular training (strength, endurance), with diet and drug restriction, and comparisons between running and the effects of environmental conditions (e.g., cold and hot).

### Outcome Measurements

Outcome measurements assessed to understand the involvement of cells, immune cells and binding molecules in short and long term exercise were: Leukocytes, neutrophils and granulocytes; NK and NKT Lymphocytes and Cells: CD3+, CD4+, CD8+, CD16+, CD18+, CD19+, CD20+, CD22+, CD44+, CD45+, CD56+, CD95+, and their proportions; Cytokines and interleukins (IL): IL-1, IL-2, IL-6, IL-8, IL-10, and IL12; Tumor Necrosis Factor (TNF-α); Interferon-Gamma (IFN-γ); Immunoglobulin (Ig): IgG, IgA, and IgM; Adhesion Molecules: ICAM-1, ICAM-2, ICAM-3 (Pedersen and Hoffman-Goetz, 2000; Nieman et al., 2001; Mooren et al., 2002; Barron et al., 2015; Brown et al., 2015; Barros et al., 2017; Koh and Park, 2018).

### Quality Assessment

The quality and risk of bias assessment of each included study was independently assessed by three authors (CAMG, MPD, IKS) using the Cochrane Risk of Bias Tool (Higgins and Green, 2011; Higgins et al., 2011; De Carvalho et al., 2013). The tool contains six domains in which each domain was classified as low, unclear or high risk of bias. Disagreements about the risk of bias assessments were resolved by consensus or by consulting the fourth author (GBCJ).

## Results

### Search Strategy

**Figure 1:**
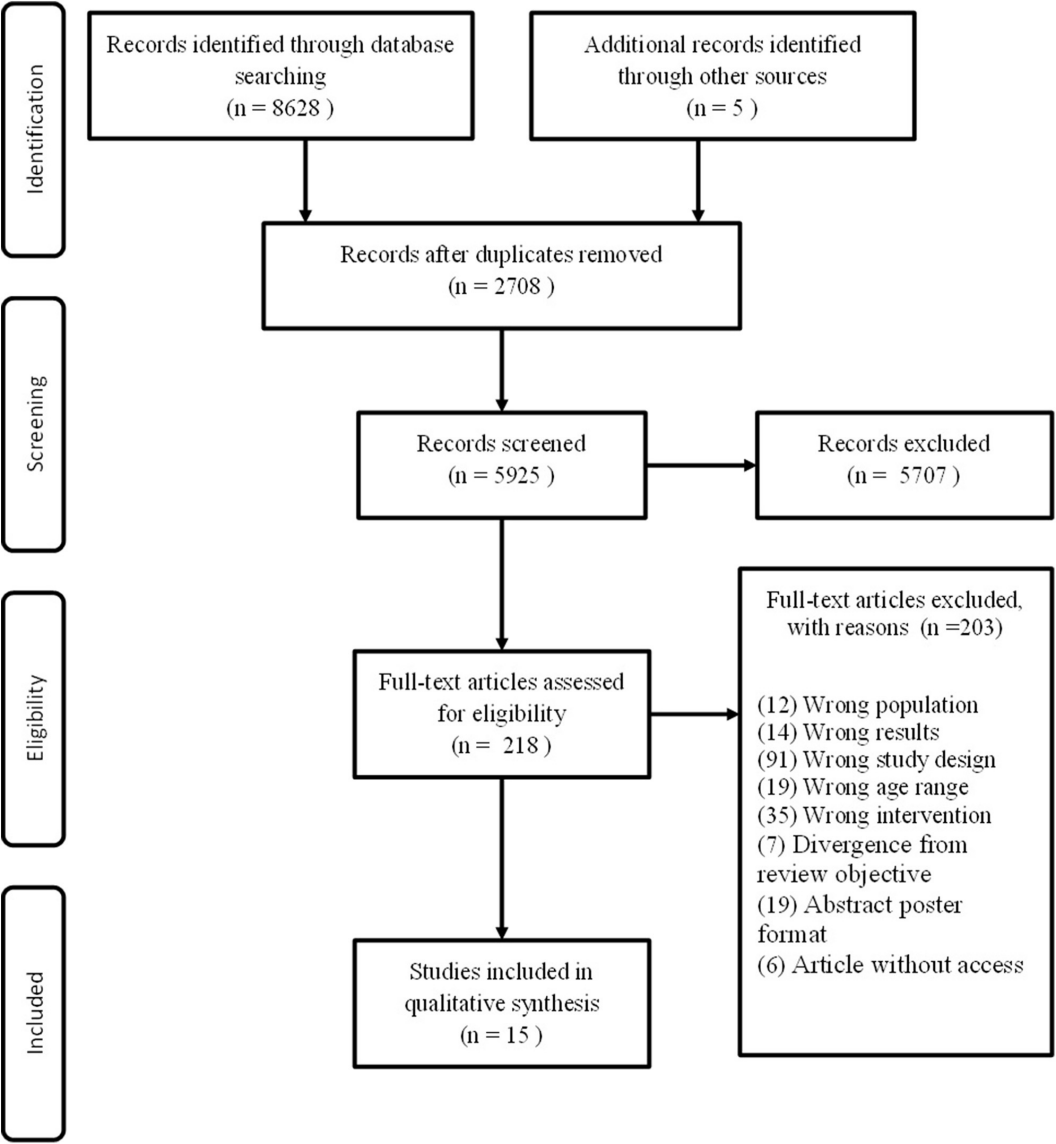
Summary of search result.

Initially, 8,633 articles were selected from the databases. After analysis, 2,708 articles were excluded because they were duplicated. 5,707 studies were then excluded by the analysis of titles and abstracts. Of the total, 218 studies had their full texts analyzed, and 203 were excluded because they were not eligible according to the inclusion criteria (Data Sheet 1_v1). After this step, 15 articles were included in this systematic review, in which 13 are related to the effect of acute exercise on immunological markers and 2 to chronic markers. The findings of the search strategy are shown in Figure 1.

### Study Features

The characteristics of the selected studies are shown in Table 1. All studies evaluated the effect of endurance exercise on the immune profile of healthy humans. Of the 15 included studies, 8 were RCTs and 7 non-RCTs, 13 were acute studies, and in two studies participants in the control and experimental groups were not the same (Nehlsen-Cannarella et al., 1991; Moyna et al., 1996a). The two studies that evaluated the chronic effect were also constituted of different individuals in the control and experimental groups (LaPerriere et al., 1994; Mitchell et al., 1996), being that 11 studies were cross-over, and 4 parallel. Cycling was performed only in 2 studies (Akerstrom et al., 2005; Scharhag et al., 2005). It was found that the practice of cycling on cycle ergometers was used as an intervention in 10 studies. While walking or running training was developed in 3 studies (Nehlsen-Cannarella et al., 1991; Green et al., 2002).

**Table 1:** Characteristics of the studies.

**References**	**Study design**	***N***	**Participants**		**Type of intervention**	**Duration (weeks)**	**Duration (min.)**	**Sessions (x/week)**	**Intensity**
			**Experimental**	**Control**					
Akerstrom et al. (2005)	Non-RCTs Cross-over	11	Men Health Age: 21–28	Same as experimental	Cycling	–	120	–	60% VO_2_máx
Edwards et al. (2006)	Non-RCTs Cross-over	24	12 Men 12 Women Health Recreational Age: 24.2 ± 3.2	Same as experimental	Cycle ergometer	–	45	–	Exercise 1: (M) 130 W (W) 95 W ↑ 35 W−3' (exhaustion) (M) 4' = 130 W (W) 4' = 95 W 45' = 55% W máx Exercise 2: (M) 16' = 84 a 231 W (W) 16' = 70 a 154 W (M) 4' =130 W (W) 4' = 95 W 25' = 55% W peak
Gabriel and Kindermann (1998)	Non-RCTs Cross-over	13	Men Health Triathletes Age: 27.5 ± 6.4	Same as experimental	Cycle ergometer	–	To exhaustion	–	110% Anaerobic Threshold
Gannon et al. (2001)	Non-RCTs Cross-over	10	Men Health Age: 26 ± 5.0	Same as experimental	Cycle ergometer	–	120	–	65% VO_2_ máx
Green et al. (2002)	RCT Cross-over	12	Men Runners Age: 30.0 ± 7.0	Same as experimental	Treadmill racing	–	60	–	95% Ventilatory Threshold
Kurokawa et al. (1995)	Non-RCTs Cross-over	8	Men Health Age: 28.5 ± 5.1	Same as experimental	Cycle ergometer	–	60	–	60% VO_2_ máx
LaPerriere et al. (1994)	RCT Parallel	14	7 Men Health Sedentary Age: 30.0 ± 6.4	7 Men Health Sedentary Age: 31.1 ± 3.1	Cycle ergometer	10	45	3	70–80% FC máx
Li and Cheng (2007)	Non-RCTs Cross-over	10	Men Health Age: 21.6 ± 0.9	Same as experimental	Cycle ergometer	–	120	–	55% VO_2_ peak
Mitchell et al. (1996)	RCT Parallel	21	11 Men Health Sedentary Age: 23.4 ± 7.0	10 Men Health Sedentary Age: 20.1 ± 1.9	Cycle ergometer	12	30	3	75% VO_2_ peak
Moyna et al. (1996a)	RCT Parallel	64	32 Adults Health 16 Men Age: 24.3 ± 0.5 16 Women Age: 23.6 ± 0.5	32 Adults Health 16 Men Age: 24.3 ± 0.5 16 Women Age: 23.6 ± 0.5	Cycle ergometer	–	18	–	55/70/85% VO_2_ peak
Moyna et al. (1996b)	RCT Parallel	64	32 Adults Health 8 Men Active Age: 24.9 ± 0.8 8 Women Active Age: 23.3 ± 0.7 8 Men Sedentary Age: 25.0 ± 0.8 8 Women Sedentary Age: 23.8 ± 0.8	32 Adults Health 8 Men Active Age: 24.9 ± 0.8 8 Women Active Age: 23.3 ± 0.7 8 Men Sedentary Age: 25.0 ± 0.8 8 Women Sedentary Age: 23.8 ± 0.8	Cycle ergometer	–	18	–	55/70/85% VO_2_ peak
Nehlsen-Cannarella et al. (1991)	RCT Cross-over	12	Women Health Age: 36.9 ± 2.2	Same as experimental	Treadmill walking	–	45	–	60% VO_2_ max
Nehlsen-Cannarella et al. (1991)	RCT Cross-over	12	Women Health Age: 36.9 ± 2.2	Same as experimental	Track walking	–	45	–	60% VO_2_ max
Ronsen et al. (2002)	RCT Cross-over	9	Men Athletes Triathletes—Skaters Age: 21–27	Same as experimental	Cycle ergometer	–	75	–	75% VO_2_ max
Scharhag et al. (2005)	Non-RCTs Cross-over	12	Men Athletes Triathletes—Cyclists Age: 26.9 ± 7.0	Same as experimental	Cycling on the running track	–	240	–	70% Anaerobic Threshold

### Participants

The total of participants in the studies were 296 healthy individuals, 196 men and 100 women. Ten studies only included men (including studies with chronic effects), 3 studies investigated men and women and just 2 studies in the included sample were women only. In the studies selected in this review, the samples were composed of triathletes and runners (Gabriel and Kindermann, 1998; Green et al., 2002; Ronsen et al., 2002; Scharhag et al., 2005), active and sedentary participants (Moyna et al., 1996b), recreational sport practitioners (Edwards et al., 2006), and sedentary individuals (LaPerriere et al., 1994; Mitchell et al., 1996). In the 5 studies that had women as participants only by Nehlsen-Cannarella et al. (1991), there was an observation about the use of oral contraceptives by women, as can be seen in Table 1.

### Intervention

Acute intervention was used in most studies included in this systematic review. The interventions performed in these observations were: cycling (Akerstrom et al., 2005; Scharhag et al., 2005), cycle ergometer (Nehlsen-Cannarella et al., 1991; Kurokawa et al., 1995; Moyna et al., 1996a; Gabriel and Kindermann, 1998; Gannon et al., 2001; Ronsen et al., 2002; Edwards et al., 2006; Li and Cheng, 2007), racing (Green et al., 2002), and walking (Nehlsen-Cannarella et al., 1991). For chronic studies (LaPerriere et al., 1994; Mitchell et al., 1996), the cycle ergometer was also used.

Prescription intensity was presented as heterogeneous, using as a parameter the percentage of VO_2_max, which ranged between 60, 65, and 75% (Nehlsen-Cannarella et al., 1991; Kurokawa et al., 1995; Gannon et al., 2001; Ronsen et al., 2002; Akerstrom et al., 2005), three studies used the VO_2_ Pico percentage (Nehlsen-Cannarella et al., 1991; Moyna et al., 1996a; Li and Cheng, 2007), two used the anaerobic threshold (Gabriel and Kindermann, 1998; Scharhag et al., 2005), one study used the ventilatory threshold (Green et al., 2002) and one used Wmax/WPeak (Edwards et al., 2006). In both studies with chronic intervention, the maximum effort used was the percentage of HRMax (LaPerriere et al., 1994) and VO_2_Peak (Mitchell et al., 1996).

To estimate workload, the intensity percentage was multiplied by the time in minutes of the intervention. Among the studies that used VO_2_max, Gannon et al. (2001) applied the highest workload (u = 7,800), followed by Akerstrom et al. (2005; u = 7,200). The lowest workloads were applied with an intensity similar to the other studies (60% VO_2_max), but with a shorter duration of 45 min (ua = 2,700; Nehlsen-Cannarella et al., 1991). With the use of VO_2_peak, 2 studies presented an incremental intensity model (55, 70, and 85%), fulfilling 6 min at each stage (ua = 1,260; Nehlsen-Cannarella et al., 1991; Moyna et al., 1996a), while one presented a continuous model with 55% for 120 min (water = 6,600; Li and Cheng, 2007).

In the 13 acute studies, 12 had the duration in their interventions range from 18 to 240 min and 1 study went to exhaustion. In chronic studies, however, different prescription parameters were set, but these can be considered moderate intensities. Regarding the duration of interventions, the data were heterogeneous, since one study lasted 10 weeks with 45-min sessions (1,350 min of exercise; LaPerriere et al., 1994), while another lasted 12 weeks with 30-min sessions (1,080 min total exercise; Mitchell et al., 1996), both studies had a training frequency of 3 times per week.

### Immunological Markers

The effect of post-aerobic regulation on the immunological markers that were evaluated in the studies are shown in Table 2. Endurance activity increased leukocytes, lymphocytes, granulocytes, neutrophils, eosinophils, and monocytes (Nehlsen-Cannarella et al., 1991; Kurokawa et al., 1995; Moyna et al., 1996a; Green et al., 2002; Scharhag et al., 2005; Li and Cheng, 2007). In the acute intervention, only one study identified no change in the amount of granulocytes and monocytes, although the leukocyte, lymphocyte, and neutrophil count was similar to other studies (Nehlsen-Cannarella et al., 1991). Other studies have shown increased CD16-56 NK cells (Nehlsen-Cannarella et al., 1991; Moyna et al., 1996a,b; Gannon et al., 2001). In regard to chronic effects, only two studies were considered valid to be included in this review. In one study it was observed that there were no changes in the amount of leukocytes, lymphocytes and monocytes, but there was an increase in CD4+, CD8+, and CD20 lymphocyte subpopulations, although no change in CD56 cells (LaPerriere et al., 1994). Similarly, Mitchell et al. (1996), found no change in the amount of lymphocytes, and neither in the amount of IgG, IgA, and IgM.

**Table 2:** Post-aerobic exercise regulation and immunological markers.

**References**	**Immune markers end regulation after exercise**																						
	**Leuc**	**Linf**	**Gran**	**Neut**	**Mon**	**Eosin**	**IL-1**	**IL-2**	**IL-6**	**IL-8**	**CD3**	**CD4**	**CD8**	**CD4 /CD8**	**CD16-56**	**CD16**	**CD56**	**CD18**	**CD19**	**CD20**	**IgG**	**IgA**	**IgM**
Akerstrom et al. (2005)										↔													
Edwards et al. (2006)									↑														
Gabriel and Kindermann (1998)																		↑					
Gannon et al. (2001)												↑	↑	↓	↑				↔				
Green et al. (2002)	↑	↑		↑	↑																		
Kurokawa et al. (1995)	↑	↑	↑									↔	↔			↑							
LaPerriere et al. (1994)	↔	↔			↔							↑	↑	↔			↔			↑			
Li and Cheng (2007)	↑	↑		↑	↑																		
Mitchell et al. (1996)		↔																			↔	↔	↔
Moyna et al. (1996a)		↑			↑						↓				↑				↓				
Moyna et al. (1996b)		↑	↑	↑	↑	↑					↑	↑	↑	↓	↑				↑				
Nehlsen-Cannarella et al. (1991)	↑	↑						↔													↑	↔	↔
Nehlsen-Cannarella et al. (1991)	↑	↑	↔	↑	↔							↔	↑		↑					↔			
Ronsen et al. (2002)							↔		↑														
Scharhag et al. (2005)	↑	↑		↑	↔			↔	↑							↑							

*Leuc, Leukocytes; Lymph, Lymphocytes; Gran, Granulocytes; Neut, Neutrophils; Mon, Monocytes, Eosn, Eosinophils (↑ increased, ↓ decreased, ↔ no change)*.

In the lymphocyte subpopulations of the immune system there are contradictory results. Two studies investigated the effect of acute exercise on CD3+ T cells, showing an increase in the count of these cells (Moyna et al., 1996b) and a reduction in the percentage of CD3+ among lymphocytes (Moyna et al., 1996a). The data on the effect on the interventions of CD4+ T helper and CD8+ cytotoxic T cells are similar. Three studies showed an increase in CD4 and CD8+ (LaPerriere et al., 1994; Moyna et al., 1996b; Gannon et al., 2001), while two showed an increase in CD8+ and unchanged CD4+ values (Nehlsen-Cannarella et al., 1991; Kurokawa et al., 1995). There is a lack of studies with the characteristics determined in the inclusion criteria of the present review that have verified the effect of exercise on CD18 cells, with only one study showing an increase in these cells (Gabriel and Kindermann, 1998). Similarly, only one study verified the effect on CD20, showing no change (Nehlsen-Cannarella et al., 1991). Data for CD19 are contradictory, with only one study presenting information on its increase (Moyna et al., 1996b), while other studies showed a reduction or lack of change in this marker (Moyna et al., 1996a; Gannon et al., 2001). Concomitantly, one study demonstrated increased IgG but no change in IgA and IgM (Nehlsen-Cannarella et al., 1991). These observations are presented in Table 2.

Few studies from those included in this review addressed the effect of aerobic exercise on interleukins. Exercise has not been found to promote IL-1 alteration (Ronsen et al., 2002), two studies have indicated that exercise does not alter IL-2 levels (Nehlsen-Cannarella et al., 1991; Scharhag et al., 2005) and just one demonstrated that IL-8 values did not change (Akerstrom et al., 2005). Three studies found increased IL-6 after an aerobic exercise session (Ronsen et al., 2002; Scharhag et al., 2005; Edwards et al., 2006). These results are expressed in Table 2.

### Quality Assessment

**Figure 2:**
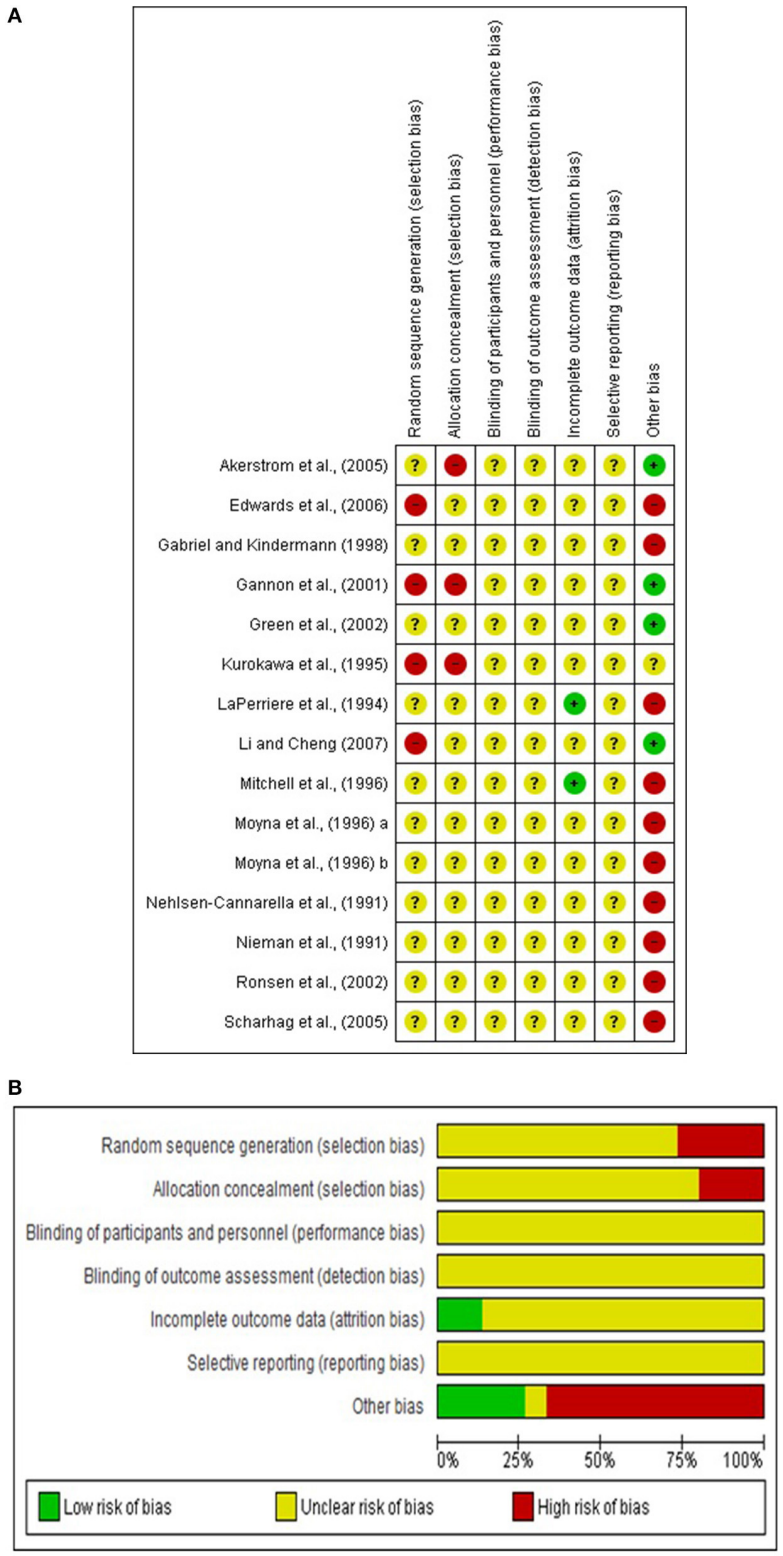
**(A,B)** Risk of bias tool.

After using the Cochrane risk of bias tool, the results are shown in Figures 2A,B.

Random sequence generation: In 11 studies no process methods were described for the generation of their random sequences, characterized as risk of unknown bias. These methods could be by generating random computer numbers, throwing coins, shuffling cards or envelopes, throwing dice or by lottery.

Allocation concealment: 12 articles in their entirety did not provide sufficient information, so, in this way, it was not possible to detect how the sequence and allocation of participants occurred.

Blindness of practitioners, participants, outcome assessors: In randomized controlled trials using exercise intervention, everyone involved cannot be blinded to treatment allocation. In the studies where supervised physical exercise was administered, the professionals who performed the intervention could not be blinded. Regarding the blindness of the outcome evaluators, this information did not exist in the evaluated studies. These facts meant that the classification of all the studies had an “unclear” risk of bias, i.e., unknown because of their lack of information.

Incomplete outcomes: In 13 studies, it was impossible to verify risk bias for losses and for the sampling stages due to the lack of described information related to randomized numbers and the reasons for losses.

Selective outcome: It can be observed that in all the studies the risk of bias for reporting a selective outcome was classified as unknown due to insufficient information in the studies. They did not have their protocols or did not allow access to them.

Other sources of bias: These sources were easily detected in 10 studies. In these there was no information relating to the nutritional status of the participants during data collection or any daily dietary report in the weeks prior to these same study evaluations.

## Discussion

The present systematic review aimed to analyze the scientific evidence on the acute and chronic effects of aerobic exercise on immune markers in healthy individuals. It was found in our study that aerobic exercise promotes changes in the immune response of leukocytes, lymphocytes, lymphocyte subpopulations, interleukins, NK cells and immunoglobulins. Several authors confirm the occurrence of these modifications in the same immunological markers due to the performance of cardiorespiratory physical training (Barron et al., 2015; Brown et al., 2015; Barros et al., 2017; Koh and Park, 2018). Therefore, the characteristics of the sample collected, such as: gender, level of fitness, sport practiced and intensity of intervention, may interfere in the values, becoming a limitation in our analysis despite the adequate comparison and generalization of the results found.

A large number of studies analyze the likely effects of aerobic exercise on immune cells by bringing together individuals of both genders in their samples (Morgado et al., 2012, 2014; Rowlands et al., 2012; Li et al., 2017). This fact limits the results found due to the existence of different sex-related immune responses in healthy individuals (Klein and Flanagan, 2016). Some female centered studies have been conducted considering the use of contraception in women (Nehlsen-Cannarella et al., 1991). There are limitations in the results found due to the use of this pharmacological therapy. These drugs directly influence leukocyte and other immune system concentrations by altering their absolute and relative numbers and may even raise them above normal values for healthy individuals (Timmons et al., 2005; Medeiros et al., 2007; Gough et al., 2015). The chronic studies used only healthy but sedentary men. This fact allows a comparative analysis of immune function with adaptation to prolonged physical training that provides an improvement in physical fitness and immune response (Sato et al., 1998; Farhangimaleki et al., 2009; Patlar, 2010; Su et al., 2011; Morgado et al., 2012).

Several authors report acute and chronic changes in immune system cells in practitioners of aerobic sports such as running, cycling, triathlon, and skating (Díaz et al., 2011; LaVoy et al., 2011; Brown et al., 2015; Santos et al., 2016; Barros et al., 2017; Koh and Park, 2018). The heterogeneity of the ways of estimating the intensity of interventions does not allow us to stipulate a better homogeneous pattern to be used. Studies show that moderate to severe aerobic exercise, whether short or long term, promotes acute and chronic changes in immune function (Pedersen, 1991; Nieman and Nehlsen-Cannarella, 1994; Pedersen and Ullum, 1994; Pedersen et al., 1998; Nieman and Pedersen, 1999; Pedersen and Hoffman-Goetz, 2000; Maltseva et al., 2011; Walsh et al., 2011; Suzuki, 2018). Acute studies with an intensity of 60% of VO_2_max during 45 and 60 min on an ergometer cycle did not cause changes in the CD4+ lymphocyte (Nehlsen-Cannarella et al., 1991; Kurokawa et al., 1995). The same intervention at 65% VO_2_max and 55/70/85% VO_2_peak increased the concentrations (Moyna et al., 1996b; Gannon et al., 2001). Therefore, changes in CD4+ are likely to be more sensitive to exercise intensity. Immunological markers such as leukocytes, lymphocytes (CD3+, CD4+, CD8+), neutrophils, monocytes, and T cells have their concentrations increased above normal values through adventure running, depending on the intensity of execution (Tossige-Gomes et al., 2014). Catecholamines and cortisol have been shown to act directly on leukocytosis during and after exercise (Mccarthy and Dale, 1998), but the intensity and volume of this activity modulate its response in the immune system. Several studies that analyze and compare the duration and intensity of interventions on the same population point to significant differences between pre and post-intervention in most of the immunological markers analyzed (Auersperger et al., 2012; Ibis et al., 2012; Witard et al., 2012; Lira et al., 2017).

Aspects related to the studies analyzed, such as the participants' level of physical fitness, type of intervention, in regard to its time and intensity, and the use of contraceptives by women may interfere with the analyzed immune cell concentrations. These possible changes in immune function bring up an important question to be answered through further investigation in future studies.

Acute aerobic exercise has been shown to be a potential influencer of immune cell concentrations, while data from chronic studies are still contradictory (Mitchell et al., 1996; Barros et al., 2017). Acute intervention influences the regulation of the immune system (Pedersen, 1991; Nieman and Nehlsen-Cannarella, 1994; Pedersen and Ullum, 1994; Pedersen et al., 1998; Nieman and Pedersen, 1999; Pedersen and Hoffman-Goetz, 2000; Maltseva et al., 2011; Walsh et al., 2011; Suzuki, 2018).

In many studies, authors report that acute aerobic exercise promotes changes in most immune markers like leukocytes, lymphocytes, and natural killer cells, among others (Scharhag et al., 2005; Patlar, 2010; Su et al., 2011; Santos et al., 2013). Long-term acute aerobic exercise with cycling, marathons, and triathlons lasting longer than multiple hours promotes elevations in plasma concentrations of IL-1ra, IL-6, IL-8, and IL-10 cytokines after exercise (Nieman et al., 2001; Ronsen et al., 2002; Suzuki, 2018). Leukocytes, neutrophils, interleukins 6, 10, 8, 12, tumor necrosis factor (TNF-α) and adhesion molecule (ICAM-1) have their plasma concentrations increased immediately after a 42.2 km marathon, enabling a post-exercise state of infection (Suzuki et al., 2003; Santos et al., 2016).

Mid-distance running (21.1 km) stimulates growth in leukocyte, neutrophil and monocyte numbers in amateur runners (Lippi et al., 2010, 2014). During a moderate 4-hour session of cycling, the absolute number of circulating NK cells, monocytes and neutrophils in the bloodstream increases, but after the exercise is finished only IL-6 concentrations remain high (Scharhag et al., 2005). Performing moderate walking for 45 min does not change T-cell (CD5 and CD25) and interleukin-2 concentrations, however, immunoglobulins have their plasma concentrations increased immediately after this walk (Nehlsen-Cannarella et al., 1991).

Lymphocyte subpopulations also increase with 10-week aerobic training, with three sessions of 45 min per week on the cycle ergometer at 70–80% intensity of the predicted maximum heart rate per age (LaPerriere et al., 1994). Corroborating with the findings of LaPerriere et al. (1994), a training session performed 3 times per week for 12 weeks, 30 min/session at 75% of VO_2_peak, also did not verify changes in total lymphocyte count (Mitchell et al., 1996). In addition, the authors also did not identify changes in IgG, IgA, and IgM immunoglobulins. The chronic effects of aerobic exercise on immunological markers are still controversial and need further investigation in further studies.

As a limitation of this study, we can highlight the difficulty of finding studies with experimental and controlled conditions. Regarding the physical fitness of the chosen study participants, which can vary from sedentary to athlete, the results cannot be generalized. In regard to gender, which is a factor of great influence on the immune system, only five articles were found from all the articles selected that investigated the female sex and in three of these both of the sexes were mixed in the sample. In regard to the intensity of physical effort used in the interventions, there is the limitation of stipulating a zone of training when considering the heterogeneity found, and that each one has an acute or chronic effect on a certain immunological marker. Another limitation of our study was the difficulty of finding studies that report on the chronic effect of exercise with only two studies being chosen for this review, thus not allowing a thorough analysis of this aspect.

In addition, the use of birth control pills in studies with women limits our ability to reach conclusions. Thus, there is a considerable need for further studies in order to identify the factors that really influence these discrepancies and also further research is needed in chronic intervention to verify its actual interference with immunological markers.

We therefore highlight the exclusivity of this review because it is the first to widely cover this subject analyzing the effects of acute and chronic aerobic exercises on all existing immunological markers. The inclusion of randomized studies, the methodological precautions adopted to reduce the risk of bias, the predefinitions of a protocol to be registered on a specific web platform, and the inclusion of experimental studies with control and experimental groups of similar characteristics helped in the observation of the true interference of aerobic exercise on immune cells. These facts show that this study has made an important contribution with great research potential within the existing scientific literature.

According to the results found in this research it was possible to understand which immune markers are affected by aerobic exercise from acute and chronic perspectives. Aspects such as the intensity of each intervention performed, its type, the place where it was developed and the characteristics of the individuals undergoing it make up important knowledge that allows for a better understanding of these immunological changes caused by aerobic exercises. This review provides an up-to-date insight into the content investigated herein, improving the safety in the work of health professionals when prescribing these aerobic exercises for healthy adults in the promotion of health.

Therefore, it is concluded from the results found in this study that acute intervention promotes changes in most immunological markers while chronic interventions influence a smaller part of them. There were differences between the results of studies with acute intervention with certain immunological markers. These may be related to the level of physical fitness of the subjects, and type and intensity of intervention.

External factors such as characteristics of the environment where the intervention was performed and its intensity and also the internal aspects related to the training time and oral contraceptive use of the participants should be taken into consideration in new studies for a better understanding of the relationship between aerobic exercise, acute and chronic interventions and their real influence on immunological markers.

## Author Contributions

CG, MD, and IS contributed to the conception and design, search, and eligibility and outcome measures. CG, MD, and GJ contributed to the quality assessment. CG, MD, IS, PD, DS, and GJ contributed to the writing of the manuscript. CG, MD, IS, PD, DS, BC, RG, and GJ contributed to the revision and approval of the final manuscript version and interpretation of the results.

### Conflict of Interest

The authors declare that the research was conducted in the absence of any commercial or financial relationships that could be construed as a potential conflict of interest.
