# Supplementary material for: Effect of Acute and Chronic Aerobic Exercise on Immunological Markers: A Systematic Review
Source: Front Physiol. 2020 Jan 24;10:1602. doi: 10.3389/fphys.2019.01602 (PMC6993577; doi:10.3389/fphys.2019.01602)
Supplement: Supplementary file 1 [file Table_1.pdf]

Table S1 : "Data Sheet 1_v1"

	TITLE ANALYZED FULL TEXT	AUTHOR/YEAR	REASON FOR EXCLUSION	DOI OR WEBSITE
1	Frequent participation in high volume exercise throughout life is associated with a more differentiated adaptive immune response	Moro-Garcia et al., (2014)	Wrong population	https://doi.org/10.1016/j.bbi.2013.12.014
2	A six-month intradialytic exercise programme has anti-inflammatory effects on circulating monocyte phenotypes and regulatory t cells but not on cytokine concentration	Dungey et al., (2015)	Wrong population	https://doi.org/10.1093/ndt/gfv197.32
3	Immune system alteration in response to increased physical training during a five day soccer training camp	Malm et al., (2004)	Wrong population	https://doi.org/10.1055/s-2004-821119
4	Immune functiom in female elite rowers and non-athletes	Nieman et al., (2000)	Wrong population	https://doi.org/10.1136/bjsm.34.3.181
5	Effects Of A Soccer Match On Biochemical Markers In Elite Male Players	Osiecki et al., (2010)	Wrong population	https://doi.org/10.1249/01.MSS.0000384812.39723.d4
6	Inhibition of interferon, cytokine, and lymphocyte proliferative responses in elite swimmers with altitude exposure	Pyne et al., (2000)	Wrong population	https://doi.org/10.1089/107999000312351
7	Changes in natural killer cell subpopulations over a winter training season in elite swimmers	Rama et al., (2012)	Wrong population	https://doi.org/10.1007/s00421-012-2490-x
8	Circulating regulatory T-cells: Acute exercise response in elite swimmers	Wilson et al., (2008)	Wrong population	https://www.fasebj.org/doi/full/10.1096/fasebj.22.1_supplement.670.19
9	Adhesion molecules during immune response to exercise	Gabriel et al., (1998)	Wrong population	https://doi.org/10.1139/cjpp-76-5-512
10	The effect of maximal exercise on the activity of neutrophil granulocytes in highly trained athletes in a moderate training period	Hack et al., (1992)	Wrong population	https://doi.org/10.1007/bf00602358
11	Role of endurance exercise in immune senescence	Nieman et al., (1994)	Wrong population	https://doi.org/10.1249/00005768-199402000-00007
12	Lymphocyte and cytokines after short periods of exercise	Prestes et al., (2008)	Wrong population	https://doi.org/10.1055/s-2008-1038737
13	Neutrophil death induced by a triathlon competition in elite athletes	Levada-Pires et al., (2008)	Wrong results	https://doi.org/10.1249/MSS.0b013e31816dc89e
14	Exercise induced mobilisation of the marginated granulocyte pool in the investigation of ethnic neutropenia	Phillips et al., (2000)	Wrong results	https://doi.org/10.1136/jcp.53.6.481
15	Exercise and the neutrophil oxidative burst: Biological and experimental variability	Pyne et al., (1996)	Wrong results	https://doi.org/10.1007/bf02376774
16	Marathon Race Affects Neutrophil Surface Molecules: Role of Inflammatory Mediators	Santos et al., (2016)	Wrong results	https://doi.org/10.1371/journal.pone.0166687

17	Expression of exercise-induced HSP70 in long-distance runner's leukocytes	Shin et al., (2004)	Wrong results	https://doi.org/10.1016/j.jtherbio.2004.08.053
18	Deleterious effects of short-term, high-intensity exercise on immune function: evidence from leucocyte mitochondrial alterations and apoptosis	Tuan et al., (2008)	Wrong results	https://doi.org/10.1136/bjsm.2006.029314
19	Warm-up exercise suppresses platelet-eosinophil/neutrophil aggregation and platelet-promoted release of eosinophil/neutrophil oxidant products enhanced by severe exercise in men	Wang et al., (2006)	Wrong results	https://doi.org/10.1160/TH05-09-0646
20	Acute effects of the cellular immune system on aerobic and anaerobic exercises	Ibis et al., (2012)	Wrong results	https://hdl.handle.net/11480/4637
21	Impact of high-intensity and high-volume exercise on short-term perturbations in the circulating fraction of different cell types	Mathes et al., (2017)	Wrong results	https://doi.org/10.23736/S0022-4707.16.05860-6
22	Acute Hematological and Inflammatory Responses to High-intensity Exercise Tests: Impact of Duration and Mode of Exercise	Minuzzi et al., (2017)	Wrong results	https://doi.org/10.1055/s-0042-117723
23	High-Intensity Training Reduces CD8(+) T-cell Redistribution in Response to Exercise	Witard et al., (2012)	Wrong results	https://doi.org/10.1249/MSS.0b013e318257d2db
24	Effect of submaximal physical exercise performed by sedentary men and women on some parameters of the immune-system	Barriga et al., (1993)	Wrong results	https://www.ncbi.nlm.nih.gov/pubmed/8378588
25	Leukocyte, lymphocyte and platelet response to dynamic exercise - Duration or intensity effect?	Gimenez et al., (1986)	Wrong results	https://doi.org/10.1007/bf00421638
26	Influence of ultra-endurance exercise on immunoglobulin isotypes and subclasses	McKune et al., (2005)	Wrong results	https://doi.org/10.1136/bjsm.2004.017194
27	Overtraining and immune system: a prospective longitudinal study in endurance athletes	Gabriel et al., (1998)	Design of the wrong study	https://doi.org/10.1097/00005768-199807000-00021
28	The effect of the tapering on the concentration of some plasma cytokines and physical performance in endurance male runners	Farhangi et al., (2010)	Design of the wrong study	https://www.sid.ir/en/journal/ViewPaper.aspx?id=172101
29	Effects of endurance exercise training on immune function	Kitao et al., (1989)	Design of the wrong study	https://www.researchgate.net/publication/293122493_Effects_of_endurance_exercise_training_on_immune_function
30	The effect of time of day and exercise on platelet functions and platelet-neutrophil aggregates in healthy male subjects	Aldemir et al., (2005)	Design of the wrong study	https://doi.org/10.1007/s11010-005-8238-8
31	High-intensity ultraendurance promotes early release of muscle injury markers	Bessa et al., (2008)	Design of the wrong study	https://doi.org/10.1136/bjsm.2007.043786
32	Airway inflammation in nonasthmatic amateur runners	Bonsignore et al., (2011)	Design of the wrong study	https://doi.org/10.1152/ajplung.2001.281.3.L668

33	The effect of strenuous exercise, calorie deficiency and sleep deprivation on white blood cells, plasma immunoglobulins and cytokines	Boyum et al., (1996)	Design of the wrong study	https://doi.org/10.1046/j.1365-3083.1996.d01-32.x
34	Effect of endurance exercise on airway cells in runners	Denguezli-Bouzgarrou et al., (2006)	Design of the wrong study	https://doi.org/10.1016/j.scispo.2005.12.001
35	Effects of a long-term training program of increasing intensity on the immune function of indoor Olympic cyclists	Ferrandez et al., (1996)	Design of the wrong study	https://doi.org/10.1055/s-2007-972900
36	Circulating mononuclear cell numbers and function during intense exercise and recovery	Field et al., (1985)	Design of the wrong study	https://doi.org/10.1152/jappl.1991.71.3.1089
37	Lymphocyte enzymatic antioxidant responses to oxidative stress following high-intensity interval exercise	Fisher et al., (2011)	Design of the wrong study	https://doi.org/10.1152/japplphysiol.00575.2010
38	A single session of intense exercise improves the inflammatory response in healthy sedentary women	Garcia et al., (2011)	Design of the wrong study	https://doi.org/10.1007/s13105-010-0052-4
39	Comparable Neutrophil Responses for Arm and Intensity-matched Leg Exercise	Leicht et al., (2017)	Design of the wrong study	https://doi.org/10.1249/MSS.0000000000001258
40	The effect of an adventure race on lymphocyte and neutrophil death	Levada-Pires et al., (2010)	Design of the wrong study	https://doi.org/10.1007/s00421-010-1363-4
41	The effect of single and repeated bouts of prolonged cycling on leukocyte redistribution, neutrophil degranulation, IL-6, and plasma stress hormone responses	Li et al., (2004)	Design of the wrong study	https://doi.org/10.1123/ijsnem.14.5.501
42	Acute variation of leucocytes counts following a half-marathon run	Lippi et al., (2010)	Design of the wrong study	https://doi.org/10.1111/j.1751-553X.2008.01133.x
43	Effects of training and taper on blood leucocyte populations in competitive swimmers: Relationships with cortisol and performance	Mujika et al., (1996)	Design of the wrong study	https://doi.org/10.1055/s-2007-972834
44	Effects of long-endurance running on immune system parameters and lymphocyte function in experienced marathoners	Nieman et al., (1989)	Design of the wrong study	https://doi.org/10.1055/s-2007-1024921
45	Lymphocyte proliferative response to 2.5 hours of running	Nieman et al., (1995)	Design of the wrong study	https://doi.org/10.1055/s-2007-973028
46	The effect of an ultra-endurance running race on mucosal and humoral immune function	Pacque et al., (2007)	Design of the wrong study	https://www.ncbi.nlm.nih.gov/pubmed/18091693
47	Changes in neutrophil surface receptor expression, degranulation, and respiratory burst activity after moderate- and high-intensity exercise	Peake et al., (2004)	Design of the wrong study	https://doi.org/10.1152/japplphysiol.01331.2003
48	Exercise-induced muscle damage, plasma cytokines, and markers of neutrophil activation	Peake et al., (2005)	Design of the wrong study	https://doi.org/10.1249/01.mss.0000161804.05399.3b

49	Exercise-induced muscle damage - effect on circulating leukocyte and lymphocyte subsets	Pizza et al., (1995)	Design of the wrong study	https://doi.org/10.1249/00005768-199503000-00012
50	Phagocytic function of blood neutrophils in sedentary young-people after physical exercise	Rodriguez et al., (1991)	Design of the wrong study	https://doi.org/10.1055/s-2007-1024680
51	Oxidative stress, inflammation, and muscle soreness in an 894-km relay trail run	Rowlands et al., (2012)	Design of the wrong study	https://doi.org/10.1007/s00421-011-2163-1
52	Leukocyte chemotactic cytokine and leukocyte subset responses during ultra-marathon running	Shin et al., (2013)	Design of the wrong study	https://doi.org/10.1016/j.cyto.2012.11.019
53	Exercise, training and neutrophil microbicidal activity	Smith et al., (1990)	Design of the wrong study	https://doi.org/10.1055/s-2007-1024788
54	No signaling in exercise training-induced anti-apoptotic effects in human neutrophils	Su et al., (2011)	Design of the wrong study	https://doi.org/10.1016/j.bbrc.2010.12.123
55	Relation between oxidative stress markers and antioxidant endogenous defences during exhaustive exercise	Sureda et al., (2005)	Design of the wrong study	https://doi.org/10.1080/10715760500177500
56	Blood cell no synthesis in response to exercise	Sureda et al., (2006)	Design of the wrong study	https://doi.org/10.1016/j.niox.2005.11.004
57	Effects of exhaustive endurance exercise and its one-week daily repetition on neutrophil count and functional status in untrained men	Suzuki et al., (1996)	Design of the wrong study	https://doi.org/10.1055/s-2007-972833
58	Impact of a competitive marathon race on systemic cytokine and neutrophil responses	Suzuki et al., (2003)	Design of the wrong study	https://doi.org/10.1249/01.MSS.0000048861.57899.04
59	Circulating cytokines and hormones with immunosuppressive but neutrophil-priming potentials rise after endurance exercise in humans	Suzuki et al., (2000)	Design of the wrong study	https://doi.org/10.1007/s004210050044
60	Different effects of exercise tests on the antioxidant enzyme activities in lymphocytes and neutrophils	Tauler et al., (2004)	Design of the wrong study	https://doi.org/10.1016/j.jnutbio.2004.03.002
61	Leukocytosis, muscle damage and increased lymphocyte proliferative response after an adventure sprint race	Tossige-Gomes et al., (2014)	Design of the wrong study	https://doi.org/10.1590/1414-431X20143187
62	Strenuous exercise - analogous to the acute-phase response	Weight et al., (1991)	Design of the wrong study	https://doi.org/10.1042/cs0810677
63	Raised plasma G-CSF and IL-6 after exercise may play a role in neutrophil mobilization into the circulation	Yamada et al., (2002)	Design of the wrong study	https://doi.org/10.1152/japplphysiol.00629.2001
64	Severe Exercise and Exercise Training Exert Opposite Effects on Human Neutrophil Apoptosis via Altering the Redox Status	Syu et al., (2011)	Design of the wrong study	https://doi.org/10.1371/journal.pone.0024385
65	Exercise-induced increase in serum interleukin-6 in humans is related to muscle damage	Bruunsgaard et al., (1997)	Design of the wrong study	https://doi.org/10.1113/jphysiol.1997.sp021972
66	Effect of brief maximal exercise on circulating levels of interleukin-12	Akimoto et al., (2000)	Design of the wrong study	https://doi.org/10.1007/s004210050076

67	Moderate endurance exercise affects hepcidin and IL-6 levels in healthy young men	Andersen et al., (2011)	Design of the wrong study	https://www.fasebj.org/doi/abs/10.1096/fasebj.25.1_supplement.607.4?related-urls=yes&legid=fasebj%3B25%2F1_Supplement%2F607.4
68	Total lymphocyte CD8 expression is not a reliable marker of cytotoxic T-cell populations in human peripheral blood following an acute bout of high-intensity exercise	Campbell et al., (2008)	Design of the wrong study	https://doi.org/10.1016/j.bbi.2007.09.001
69	Effect of a 2000-m running test on antioxidant and cytokine response in plasma and circulating cells	Carrera-Quintanar et al., (2017)	Design of the wrong study	https://doi.org/10.1007/s13105-017-0575-z
70	A single session of intense exercise improves the inflammatory response in healthy sedentary women	Garcia et al., (2011)	Design of the wrong study	https://doi.org/10.1007/s13105-010-0052-4
71	Short and medium-term influence of physical activity on immune parametersa	Grazzi et al., (1993)	Design of the wrong study	https://doi.org/10.3109/00207459309000609
72	Changes in peripheral-blood lymphocyte subsets associated with marathon running	Haq et al., (1993)	Design of the wrong study	https://doi.org/10.1249/00005768-199302000-00004
73	Exercise-induced change in type 1 cytokine-producing CD8(+) T cells is related to a decrease in memory T cells	Ibfelt et al., (2002)	Design of the wrong study	https://doi.org/10.1152/japplphysiol.01214.2001
74	Body composition, maximal aerobic performance and inflammatory biomarkers in endurance-trained athletes	Jurimae et al., (2015)	Design of the wrong study	https://doi.org/10.1111/cpf.12299
75	Acute exercise alters the Type 1/Type 2 cytokine balance in both low (CD27+) and highly (CD27-) differentiated subsets of CD4+and CD8+T cells	LaVoy et al., (2011)	Design of the wrong study	https://www.jimmunol.org/content/186/1_Supplement/117.23
76	Change in the red blood cell immunity function and T-lymphocyte and its subpopulations before and after acute incremental load exercise	Li et al., (2017)	Design of the wrong study	https://www.alliedacademies.org/articles/change-in-the-red-blood-cell-immunity-function-and-tlymphocyte-and-its-subpopulations-before-and-after-acute-incremental-load-exer-8123.html
77	Effects of high- vs moderate-intensify exercise on natural killer cell activity	Nieman et al., (1993)	Design of the wrong study	https://doi.org/10.1249/00005768-199310000-00008
78	Effects of acute and 4-week submaximal exercise on leukocyte and leukocyte subgroups	Patlar et al., (2010)	Design of the wrong study	https://doi.org/10.3233/IES-2010-0373
79	The Relationship Between Maximal Exercise-Induced Increases in Serum IL-6, MPO and MMP-9 Concentrations	Reihmane et al., (2012)	Design of the wrong study	https://doi.org/10.1111/j.1365-3083.2012.02720.x
80	The effect of repeated endurance exercise on IL-6 and sIL-6R and their relationship with sensations of fatigue at rest	Robson-Ansley et al., (2009)	Design of the wrong study	https://doi.org/10.1016/j.cyto.2008.11.006

81	Bicycle exercise enhances plasma il-6 but does not change il-1-alpha, il-1-beta, il-6, or tnf-alpha pre-messenger-rna in bmnc	Ullum et al., (1994)	Design of the wrong study	https://doi.org/10.1152/jappl.1994.77.1.93
82	Effect of exhaustive exercise stress on the cytokine response	Weinstock et al., (1997)	Design of the wrong study	https://doi.org/10.1097/00005768-199703000-00009
83	Increased inflammatory response of blood cells to repeated bout of endurance exercise	Degerstrom et al., (2006)	Design of the wrong study	https://doi.org/10.1249/01.mss.0000227315.93351.8d
84	Effects of brief maximal exercise on interleukiin-6 and tumor necrosis factor-alpha	Denguezli-Bouzgarrou et al., (2006)	Design of the wrong study	https://www.researchgate.net/publication/233808434_Effects_of_brief_maximal_exercise_on_interleukin-6_and_tumor_necrosis_factor-alpha
85	Exercise and the Regulation of Inflammatory Responses	Allen et al., (2015)	Design of the wrong study	https://doi.org/10.1016/bs.pmbts.2015.07.003
86	Evidence for an exercise induced increase of TNF- and IL-6 in marathon runners	Bernecker et al., (2011)	Design of the wrong study	https://doi.org/10.1111/j.1600-0838.2011.01372.x
87	Effect of indoor climbing exercise on plasma oxidative stress, hematologic parameters and heart rate responses in sedentary individuals	Cesur et al., (2012)	Design of the wrong study	https://www.alliedacademies.org/articles/effect-of-indoor-climbing-exercise-on-plasma-oxidative-stress-hematologic-parameters-and-heart-rate-responses-in-sedentary-individ.html
88	T-regulatory cells exhibit a biphasic response to prolonged endurance exercise in humans	Clifford et al., (2017)	Design of the wrong study	https://doi.org/10.1007/s00421-017-3667-0
89	Immunological responses to four-day of consecutive and non-consecutive circuit resistance exercise	Mohebbi et al., (2012)	Design of the wrong study	https://www.minervamedica.it/en/journals/medicina-dello-sport/article.php?cod=R26Y2012N04A0485
90	Effects of chronic intense exercise training on the leukocyte response to acute exercise	Ndon et al., (1992)	Design of the wrong study	https://doi.org/10.1055/s-2007-1021252
91	The impact of prolonged strenuous endurance exercise on interleukin 18 and interleukin 18 binding protein in recreational cyclists	Neumayr et al., (2005)	Design of the wrong study	https://doi.org/10.1055/s-2005-837466
92	Influence of physical-activity on the cellular immune-system - mechanisms of action	Pedersen et al., (1991)	Design of the wrong study	https://doi.org/10.1055/s-2007-1024746
93	The impact of soccer training on the immune system	Rebelo et al., (1998)	Design of the wrong study	https://www.ncbi.nlm.nih.gov/pubmed/9830835
94	The impact of exercise on the immune system: NK cells, interleukins 1 and 2, and related responses	Shephard et al., (1995)	Design of the wrong study	https://doi.org/10.1249/00003677-199500230-00009
95	Increased immune activation during and after physical excercise	Tilz et al., (1993)	Design of the wrong study	https://doi.org/10.1016/S0171-2985(11)80497-3

96	Assessment of oxidative stress in lymphocytes with exercise	Turner et al., (2011)	Design of the wrong study	https://doi.org/10.1152/japplphysiol.00051.2011
97	Immunological Status of Competitive Cyclists Before and After the Training Season	Baj et al., (1994)	Design of the wrong study	https://doi.org/10.1055/s-2007-1021067
98	White blood cell counts in elite triathletes over four consecutive seasons	Díaz et al., (2011)	Design of the wrong study	https://doi.org/10.1007%2Fs00421-010-1701-6
99	Changes in blood leucocyte populations induced by acute maximal and chronic submaximal exercise	Ferry et al;. (1990)	Design of the wrong study	https://doi.org/ 10.1007/bf02388625
100	Immune Function in Athletes Versus Nonathletes	Nieman et al., (1995)	Design of the wrong study	https://doi.org/10.1055/s-2007-973014
101	Run training versus cross-training: effect of increased training on circulating leukocyte subsets	Pizza et al., (1995)	Design of the wrong study	https://www.ncbi.nlm.nih.gov/pubmed/7752862
102	Long Term Swimming Training Influence On The Immune Response To High Intensity Sessions	Morgado et al., (2016)	Design of the wrong study	https://doi.org/10.1249/01.mss.0000488093.09976.f0
103	Immune function in marathon versus sedentary controls	Nieman et al., (1995)	Design of the wrong study	https://doi.org/10.1249/00005768-199507000-00006
104	Effect of acute exercise on some haematological parameters and neutrophil functions in active and inactive subjects	Benoni et al., (1995)	Design of the wrong study	https://doi.org/10.1007/bf00361548
105	The impact of 6-month training preparation for an Ironman triathlon on the proportions of naive, memory and senescent T cells in resting blood	Cosgrove et al. (2011)	Design of the wrong study	https://doi.org/10.1007/s00421-011-2273-9
106	Effect of Selected Exercise on Serum Immunoglobulin (IgA , IgG , and IgM) In Middle-Endurance Elite Runners	Hejazi et al., (2012)	Design of the wrong study	https://www.researchgate.net/publication/233990638_Effect_of_Selected_Exercise_on_Serum_Immunoglobulin_IgA_IgG_and_IgM_In_Middle-Endurance_Elite_Runners
107	Effects of acute exhaustive exercise and chronic exercise training on type 1 and type 2 T lymphocytes	Lancaster et al., (2004)	Design of the wrong study	https://insights.ovid.com/pubmed?pmid=15633589&clickthrough=y
108	Induction of lymphocyte death by short- and long-duration triathlon competitions	Levada-Pires et al., (2009)	Design of the wrong study	https://doi.org/10.1249/MSS.0b013e3181a327a2
109	Sex-based effects on immune changes induced by a maximal incremental exercise test in well-trained swimmers	Morgado et al., (2014)	Design of the wrong study	https://www.ncbi.nlm.nih.gov/pmc/articles/PMC4126313/
110	Hematological responses para training e taper in competitive swimmers: relationships with performance	Mujika et al., (1997)	Design of the wrong study	https://www.ncbi.nlm.nih.gov/pubmed/9711359
111	Effect of high- versus moderate-intensity exercise on lymphocyte subpopulations and proliferative response	Nieman et al., (1994)	Design of the wrong study	https://doi.org/10.1055/s-2007-1021047

112	Acute Changes in Inflammatory Biomarker Levels in Recreational Runners Participating in a Marathon or Half-Marathon	Niemelä et al., (2016)	Design of the wrong study	https://doi.org/10.1186/s40798-016-0045-0
113	Intestinal IgA- and IgM-producing cells are not decreased in marathon runners	Nilssen et al., (1998)	Design of the wrong study	https://doi.org/10.1055/s-2007-971940
114	Serum from exercising humans suppresses t-cell cytokine production	Radom-Aizik et al., (2007)	Design of the wrong study	https://doi.org/10.1016/j.cyto.2007.08.008
115	Strenuous exercise and immunological changes: A multiple-time-point analysis of leukocyte subsets, CD4/CD8 ratio, immunoglobulin production and NK cell response	Shek et al., (1995)	Design of the wrong study	https://doi.org/10.1055/s-2007-973039
116	Acute Exercise and Immune Function: Relationship between Lymphocyte Activity and Changes in Subset Counts	Shinkai et al., (1992)	Design of the wrong study	https://doi.org/10.1055/s-2007-1021297
117	Immune responses and increased training of the elite athlete Immune responses and increased training of the elite athlete	Verde et al., (1992)	Design of the wrong study	https://doi.org/10.1152/jappl.1992.73.4.1494
118	Influence of Vigorous Training on Innate Immunity in Young Athletes	Tsybulkina et al., (2017)	Wrong age range	https://doi.org/10.1016/j.jaci.2016.12.366
119	A competitive marathon race decreases neutrophil functions in athletes	Chinda et al., (2003)	Wrong age range	https://doi.org/10.1002/bio.744
120	Association of white blood cell subfraction concentration with fitness and fatness	Johannsen et al., (2010)	Wrong age range	https://doi.org/10.1136/bjsm.2008.050682
121	Mean Platelet Volume (MPV) Predicts Middle Distance Running Performance	Lippi et al., (2014)	Wrong age range	https://doi.org/10.1371/journal.pone.0112892
122	Effects of maximal exercise on nonspecific immunity in athletes under trained and detrained conditions	MochizukI et al., (1999)	Wrong age range	https://doi.org/10.7600/jspfsm1949.48.147
123	Cytokine production by monocytes, neutrophils, and dendritic cells is hampered by long-term intensive training in elite swimmers	Morgado et al., (2012)	Wrong age range	https://doi.org/10.1007/s00421-011-1966-4
124	Effects of chronic intense exercise training on the leukocyte response to acute exercise	Ndon et al., (1992)	Wrong age range	https://doi.org/10.1055/s-2007-1021252
125	Differential white cell count after two bouts of downhill running	Smith et al., (1998)	Wrong age range	https://doi.org/10.1055/s-2007-971941
126	Effects of exercise intensity on circulating leukocyte subpopulations	Saito et al., (2003)	Wrong age range	https://doi.org/10.1007/BF02897939
127	Endurance exercise causes interaction among stress hormones, cytokines, neutrophil dynamics, and muscle damage	Suzuki et al., (1999)	Wrong age range	https://doi.org/10.1152/jappl.1999.87.4.1360
128	Training status and sex influence on senescent T-lymphocyte redistribution in response to acute maximal exercise	Brown et al., (2014)	Wrong age range	https://doi.org/10.1016/j.bbi.2013.10.031
129	Strenuous exercise decreases the percentage of type 1 T cells in the circulation	Steensberg et al., (2001)	Wrong age range	https://doi.org/10.1152/jappl.2001.91.4.1708

130	Physical fitness attenuates leukocyte-endothelial adhesion in response to acute exercise	Mills et al., (2006)	Wrong age range	https://doi.org/10.1152/japplphysiol.00135.2006
131	Effekt einer aeroben ausdauerbelastung auf die Immunfunktion bei Aterssportlern	Bauer et al., (2002)	Wrong age range	https://doi.org/10.1024/0369-8394.91.5.153
132	Cytokine response to strenuous exercise in athletes and non-athletes-an adaptive response	Gokhale et al., (2007)	Wrong age range	https://doi.org/10.1016/j.cyto.2007.08.006
133	Routine exercise alters measures of immunity and the acute phase reaction	Horn et al., (2014)	Wrong age range	https://doi.org/10.1007/s00421-014-3028-1
134	Effect of Marathon Running on Hematologic and Biochemical Laboratory Parameters, Including Cardiac Markers	Kratz et al., (2002)	Wrong age range	https://doi.org/10.1309/14TY-2TDJ-1X0Y-1V6V
135	Plasma Cytokine Profiles in Long-Term Strenuous Exercise	Nielsen et al., (2016)	Wrong age range	https://doi.org/10.1155/2016/7186137
136	Changes of Hematological Markers during a Multi-stage Ultra-marathon Competition in the Heat	Rama et al., (2015)	Wrong age range	https://doi.org/10.1055/s-0035-1555929
137	Plasma Interleukin-6 Response to Environmental Temperature with Endurance Exercise	Dinan et al., (2017)	Wrong intervention	https://doi.org/10.1249/01.mss.0000517644.91711.17
138	Lymphocyte-proliferation responses after exercise in men - fitness, intensity, and duration effects	Macneil et al., (1991)	Wrong intervention	https://doi.org/10.1152/jappl.1991.70.1.179
139	Leukocytes, lymphocytes, activation parameters and cell-adhesion molecules in middle-distance runners under different training conditions	Baum et al., (1994)	Wrong intervention	https://doi.org/10.1055/s-2007-1021126
140	Effects of acute exercise on lymphocyte subsets and metabolic-activity	Frisina et al., (1994)	Wrong intervention	https://doi.org/10.1055/s-2007-1021017
141	The influence of blood volume changes on leucocyte and lymphocyte subpopulations in elite swimmers following interval training of varying intensities	Kargotich et al., (1997)	Wrong intervention	https://doi.org/10.1055/s-2007-972649
142	Differences in metabolic and inflammatory responses in lower and upper body high-intensity intermittent exercise	Lira et al., (2015)	Wrong intervention	https://doi.org/10.1007/s00421-015-3127-7
143	Leukocyte counts and lymphocyte responsiveness associated with repeated bouts of strenuous endurance exercise	Ronsen et al., (2001)	Wrong intervention	https://doi.org/10.1152/jappl.2001.91.1.425
144	mmunometabolic Responses after Short and Moderate Rest Intervals to Strength Exercise with and without Similar Total Volume	Agostinete et al., (2016)	Wrong intervention	https://doi.org/10.3389/fphys.2016.00444
145	Changes of immune system in military recruits after the training program	Anomasiri et al., (2002)	Wrong intervention	https://www.ncbi.nlm.nih.gov/pubmed/12188431
146	Immune responses to an upper body tri-set resistance training session	Brunelli et al., (2013)	Wrong intervention	https://doi.org/10.1111/cpf.12066

147	Effects of 8-Week Hatha Yoga Training on Metabolic and Inflammatory Markers in Healthy, Female Chinese Subjects: A Randomized Clinical Trial	Chen et al., (2016)	Wrong intervention	https://doi.org/10.1155/2016/5387258
148	The acute response of neutrophil function to a bout of judo training	Chinda et al., (2003)	Wrong intervention	https://doi.org/10.1002/bio.739
149	Acute response of peripheral CCr5 chemoreceptor and NK cells in individuals submitted to a single session of low-intensity strength exercise with blood flow restriction	Dorneles et al., (2016)	Wrong intervention	https://doi.org/10.1111/cpf.12231
150	Circuit resistance training in sedentary women: body composition and serum cytokine levels	Ferreira et al., (2010)	Wrong intervention	https://doi.org/10.1139/H09-136
151	Effects of resistance training at different loads on inflammatory markers in young adults	Forti et al., (2017)	Wrong intervention	https://doi.org/10.1007/s00421-017-3548-6
152	Immunological Patterns during Regular Intensive Training in Athletes: Quantification and Evaluation of a Preventive Pharmacological Approach	Garagiola et al., (1995)	Wrong intervention	https://doi.org/10.1177/030006059502300201
153	Acute leukocyte, cytokine and adipocytokine responses to maximal and hypertrophic resistance exercise bouts	Ihalainen et al., (2014)	Wrong intervention	https://doi.org/10.1007/s00421-014-2979-6
154	Effect of passive repetitive isokinetic training on cytokines and hormonal changes	Lee et al., (2011)	Wrong intervention	https://doi.org/10.4077/CJP.2011.AMM086
155	Effects of eccentric exercise on the immune system in men	Malm et al., (1999)	Wrong intervention	https://doi.org/10.1152/jappl.1999.86.2.461
156	Strength, workload, anaerobic intensity and the immune response to resistance exercise in women	Miles et al., (2003)	Wrong intervention	https://doi.org/10.1046/j.1365-201X.2003.01124.x
157	Evidence that interleukin-6 is produced in human skeletal muscle during prolonged running	Ostrowski et al., (1998)	Wrong intervention	https://doi.org/10.1111/j.1469-7793.1998.949bp.x
158	Time Course of Leukocyte Accumulation in Human Muscle after Eccentric Exercise	Paulsen et al., (2010)	Wrong intervention	https://doi.org/10.1249/MSS.0b013e3181ac7adb
159	Acute impact of submaximal resistance exercise on immunological and hormonal parameters in young men	Ramel et al., (2003)	Wrong intervention	https://doi.org/10.1080/02640410310001641395
160	Effect of qigong training on proportions of t-lymphocyte subsets in human peripheral-blood	Ryu et al., (1995)	Wrong intervention	https://doi.org/10.1142/S0192415X95000055
161	Interleukin-6 expression after repeated bouts of eccentric exercise	Willoughby et al., (2003)	Wrong intervention	https://doi.org/10.1055/s-2003-37197
162	Differences in the inflammatory plasma cytokine response following two elite female soccer games separated by a 72-h recovery	Andersson et al., (2010)	Wrong intervention	https://doi.org/10.1111/j.1600-0838.2009.00989.x
163	Immune response to exercise in elite sportsmen during the competitive season	Córdova et al., (2010)	Wrong intervention	https://doi.org/10.1007/s13105-010-0001-2

164	Effect of elite cycling on leucocyte counts	Lesesve et al., (2000)	Wrong intervention	https://doi.org/10.1046/j.1365-2141.2000.02270-6.x
165	Investigating the cellular and metabolic responses of world-class canoeists training: A sportomics approach	Coelho et al. (2016)	Wrong intervention	https://doi.org/10.3390/nu8110719
166	Effects of Resistance or Aerobic Exercise Training on Interleukin-6, C-Reactive Protein, and Body Composition	Donges et al., (2010)	Wrong intervention	https://doi.org/10.1249/MSS.0b013e3181b117ca
167	Metabolic and anti-inflammatory benefits of eccentric endurance exercise - A pilot study	Drexel et al., (2008)	Wrong intervention	https://doi.org/10.1111/j.1365-2362.2008.01937.x
168	The immediate leukocytosis to anaerobic exercise : Evidenc of two subsequent phases	Gabriel et al., (2000)	Wrong intervention	https://www.researchgate.net/publication/263843730_The_immediate_leukocytosis_to_anaerobic_exercise_Evidenc_of_two_subsequent_phases
169	Aerobik ve anaerobik egzersizlerin hematolojik parametrelere akut etkisi	Ibis et al., (2010)	Wrong intervention	http://www.acarindex.com/dosyalar/makale/acarindex-1423936624.pdf
170	Effects of a simulated tennis match on lymphocyte subset measurements	Schafer et al., (2014)	Wrong intervention	https://doi.org/10.1080/02701367.2013.872219
171	Inflammatory markers CD11b, CD16, CD66b, CD68, myeloperoxidase and neutrophil elastase in eccentric exercised human skeletal muscles	Paulsen et al., (2012)	Wrong intervention	https://doi.org/10.1007/s00418-012-1061-x
172	Investigation of the effect of marathon running on leucocyte counts of subjects of different ethnic origins: relevance to the aetiology of ethnic neutropenia	Bain et al., (2000)	Divergence from review objective	https://doi.org/10.1046/j.1365-2141.2000.01922.x
173	Effects of acute stress on lymphocyte-b(2)-adrenoceptors in white males	Chi et al., (1993)	Divergence from review objective	https://doi.org/10.1016/0022-3999(93)90105-O
174	Effects of acute endurance exercise and 8 week training on the production of reactive oxygen species from neutrophils in untrained men	Sato et al., (1998)	Divergence from review objective	https://doi.org/10.1265/jjh.53.431
175	Peripheral blood leucocyte functional responses to acute eccentric exercise in humans are influenced by systemic stress, but not by exercise-induced muscle damage	Saxton et al., (2003)	Divergence from review objective	https://www.ncbi.nlm.nih.gov/pubmed/12519089
176	Active recovery and post-exercise white blood cell count, free fatty acids, and hormones in endurance athletes	Wigernaes et al., (2001)	Divergence from review objective	https://doi.org/10.1007/s004210000365
177	Effect of acute and chronic submaximal exercise on plasma renin and aldosterone levels in football players	Patlar et al., (2011)	Divergence from review objective	https://doi.org/10.3233/IES-2011-0428
178	The effect of a 100-km ultra-marathon under freezing conditions on selected immunological and hematological parameters	Žákovská, A., et al. (2017). "	Divergence from review objective	https://doi.org/10.3389/fphys.2017.00638

179	Immune Responses To An Acute Maximal Exercise Changes During A Training Cycle In Swimming	Alves et al., (2010)	Abstract poster format	https://doi.org/10.1249/01.MSS.0000400933.36840.8b
180	Immune And Inflammatory Responses And Exercise Performance During 135 Miles Mountain Foot Race	Belli et al., (2011)	Abstract poster format	https://doi.org/10.1249/01.MSS.0000402154.10018.05
181	The Effects Of Exercise Intensity And Duration On NK-cell Cytotoxicity And Proliferative Responses To IL-15	Bigley et al., (2016)	Abstract poster format	https://doi.org/10.1249/01.mss.0000485268.23662.56
182	Effects Of Different Exercise Stimuli On Leukocyte Subsets	Carlson et al., (2008)	Abstract poster format	https://doi.org/10.1249/01.mss.0000322838.27024.79
183	Changes In Neutrophil Function After A Marathon Race	Cury-Boaventura et al., (2010)	Abstract poster format	https://doi.org/10.1249/01.MSS.0000385807.14218.c2
184	Maximal Exercise Significantly Affects Helper T-Cell (CD3+/CD4+) Count Compared to Rest and 1-h Post Exercise	Kell et al., (2010)	Abstract poster format	https://doi.org/10.1249/01.MSS.0000385805.06594.e4
185	The effects of a 16 week aerobic exercise programme on circulating lymphocyte subpopulations: A randomised controlled trial	Lavelle et al., (2015)	Abstract poster format	https://doi.org/10.1016/j.physio.2015.03.1657
186	Effects of Submaximal Downhill Running on Plasma Cytokine Expression in Young, Endurance Trained Individuals	Macko et al., (2017)	Abstract poster format	https://doi.org/10.1249/01.mss.0000519446.02920.62
187	The Frequency Of Highly Differentiated And Senescent Blood T-cells Following Two Different Endurance Training Programmes	Neal et al., (2011)	Abstract poster format	https://doi.org/10.1249/01.MSS.0000401340.02439.9c
188	The Effect Of An Adventure Sprint Race In The Haematological Parameters Of Athletes	Ottone et al., (2011)	Abstract poster format	https://doi.org/10.1249/01.mss.0000401561.28068.91
189	The Impact of Ironman Triathlon on Innate Immune Cell Numbers and Function	Radom-Aizik et al., (2013)	Abstract poster format	https://kins.uconn.edu/person/elaine-choung-hee-lee/
190	The Effect Of The Training-competition Phases In Immune Response In Triathlon	Rangel-Colmenero et al., (2012)	Abstract poster format	http://eprints.uanl.mx/4795/
191	Reduced frequency of regulatory T cells in competitive athletes	Rogosch et al., (2012)	Abstract poster format	https://insights.ovid.com/crossref?an=00004227-201209001-00631&clickthrough=y
192	Cytokine Responses To Strenuous Exercise In Elite Athletes And Sedentary Subjects	Rongen et al., (2008)	Abstract poster format	https://doi.org/10.1249/01.mss.0000322837.19401.27
193	Effect Of An Adventure Race In The Profile Of Peripheral Blood Leukocytes Of Athletes	Sampaio et al., (2011)	Abstract poster format	https://doi.org/10.1249/01.MSS.0000401556.97572.ca
194	Changes in Adhesion Molecule Expression on Natural Killer Cell Subsets during Incremental Exercise	Suzui et al., (2008)	Abstract poster format	https://doi.org/10.1249/01.mss.0000322834.34648.4d
195	Shifting gears: Immune system parameters of middle-aged marathon runners versus sedentary controls	Thomas et al., (2012)	Abstract poster format	https://insights.ovid.com/crossref?an=00004227-201209001-01051&clickthrough=y

196	Effects Of A Three-day Period Of Intense, Intermittent Exercise On Oxidative Stress And Inflammation	Holland et al, (2016)	Abstract poster format	https://doi.org/10.1249/01.mss.0000486187.56329.39
197	Acute Resistance Exercise does not Increase Skeletal Muscle Fibroblast Content in Young, Untrained Men	Doty et al., (2005)	Abstract poster format	https://doi.org/10.1097/00005768-200505001-01255
198	Changes in the number of leukocytes and lymphocyte subpopulations induced by exercise in sedentary young-people	Barriga et al., (1990)	Article without access	https://doi.org/https://insights.ovid.com/pubmed?pmid=2274706&clickthrough=y
199	Effects of a single incremental exhausting exercise on circulting numbers of lymplocyes subsets in male athletes	Far et al., (2011)	Article without access	http://eprints.ajums.ac.ir/id/eprint/18976
200	Physical activity both enhances and depresses the immune system	Malm et al., (2005)	Article without access	http://umu.diva-portal.org/smash/record.jsf?pid=diva2%3A217506&dswid=mainwindow
201	Exercise-induced generation of interleukin-1 and interleukin-2	Smith et al., (1990)	Article without access	sem endereço eletrónico
202	Effects Of Acute Resistance Exercise On Differential Leukocytes And Serum Cortisol	Carlson et al., (2005)	Article without access	https://doi.org/10.1097/00005768-200505001-01939
203	Surface ultrastructural changes in human leucocytes following submaximal exercise	Ferguson et al., (1996)	Article without access	https://www.researchgate.net/publication/297573485_Surface_ultrastructural_changes_in_human_leucocytes_following_submaximal_exercise
